# The Financial Toll of Social Determinants of Health on Orthopedic Trauma Care: A National Perspective

**DOI:** 10.7759/cureus.89212

**Published:** 2025-08-01

**Authors:** Anuja L Sarode, Amber Kerstetter-Fogle, Nathan R Blecker

**Affiliations:** 1 Surgery and Trauma, Summa Health System, Akron, USA; 2 Neurology, Summa Health System, Akron, USA; 3 Surgery, Northeast Ohio Medical University, Rootstown, USA; 4 Surgery, Summa Health System, Akron, USA

**Keywords:** health disparities, hospitalization cost, injury prevention, medicaid, mental health, nis, orthopedic trauma, social determinants of health, substance use

## Abstract

Background: Social determinants of health (SDoH) are associated with increased healthcare costs across various conditions, yet their impact on orthopedic trauma remains understudied. Orthopedic injuries impose a significant financial burden on the healthcare system, but the extent to which SDoH contribute to hospitalization costs is not well established. This study evaluates the association between documented SDoH-related diagnoses and inpatient costs in orthopedic trauma using a nationally representative dataset.

Methods: A retrospective cross-sectional study of the 2016-2021 National Inpatient Sample (NIS) was conducted. Fracture-related hospitalizations were identified using the Clinical Classifications Software Refined (CCSR) for the ICD-10: International Classification of Diseases, 10th Revision (ICD-10)-CM diagnoses (INJ001-INJ006, INJ038-INJ043). The primary outcome, hospitalization cost, was adjusted to 2024 dollars using consumer price index (CPI) data. SDoH presence was determined via ICD-10 Z-codes (CCSR group FAC019). Multivariate linear regression, adjusted for demographic and clinical factors, examined cost differences between patients with and without SDoH-related diagnoses.

Results: A total of 1,160,566 orthopedic trauma admissions were analyzed. Patients with documented SDoH factors were younger (58 vs. 72 years; p<0.001), more likely to be Black (14.02% vs. 8.07%; p<0.001), and from the lowest income quartile (36.23% vs. 27.91%; p<0.001). They had longer hospital stays (five vs. four days; p<0.001), more ED visits (83.83% vs. 79.82%; p<0.001), and higher mean costs ($27,025 vs. $22,915; p<0.001), contributing to $26.7 billion in total costs. Compared to patients without SDoH factors, they had fewer hip fractures (23.48% vs. 35.01%; p<0.001), but more upper extremity (18.70% vs. 16.56%; p<0.001) and lower extremity fractures excluding the hip (28.27% vs. 24.40%; p<0.001). Assault-related (9.76% vs. 1.60%; p<0.001) and firearm-related injuries (2.36% vs. 0.99%; p<0.001) were also more frequent. Comorbidities were disproportionately higher, including alcohol use (23.99% vs. 6.66%; p<0.001), opioid use (7.73% vs. 2.97%; p<0.001), tobacco use (39.35% vs. 15.55%; p<0.001), and schizophrenia (8.22% vs. 1.32%; p<0.001). Key cost drivers included length of stay, procedures, and substance use. Among patients with SDoH-related diagnoses, opioid use increased costs by 6.0% (vs. 7.1% in non-SDoH), stimulant use by 9.5% (vs. 6.2%), and alcohol use by 3.2% (vs. 4.4%) (all p<0.001).

Conclusion: Patients with documented SDoH-related diagnoses face significantly higher hospitalization costs in orthopedic trauma, driven by longer stays, greater comorbidity burden, and behavioral health conditions. Findings support the need for multi-sector strategies, including Medicaid reform, targeted prevention, and integration of social risk and behavioral health services in trauma care.

## Introduction

Social determinants of health (SDoH) play a critical role in shaping health outcomes, healthcare utilization, and disparities in medical conditions, including orthopedic injuries [1-5]. Factors such as socioeconomic status, education, employment, housing stability, and social environment not only affect the risk of sustaining orthopedic injuries but also impact the quality and cost of care received. Individuals with lower income, inadequate housing, or limited access to healthcare resources may face increased exposure to occupational hazards, environmental risks, and delayed treatment for fractures [6,7]. These disparities in access and care contribute to differences in injury severity, recovery time, healthcare cost, and overall health outcomes [8,9].

Orthopedic trauma exerts a substantial financial strain on the healthcare system, with estimated lifetime costs exceeding $326 billion in productivity loss and $80 billion in direct healthcare expenses in 2006 [3,10,11]. In 2023 alone, fractures of the lower limb accounted for over $375 million in hospitalization costs, representing approximately 2% of total national healthcare expenditures [12]. Similarly, the hospitalization costs associated with rib fractures exceeded $469 million in 2016 [13], underscoring the substantial economic impact of fracture-related injuries on the healthcare system. The financial burden extends beyond initial hospital stays, encompassing surgical interventions, rehabilitation, and long-term care, making fractures one of the most expensive conditions treated in US hospitals [14,15]. Prior research has consistently shown that SDoH factors, such as lower income, inadequate insurance coverage, and residence in medically underserved areas, exacerbate these costs by limiting access to timely and appropriate care. Delayed treatment due to financial or logistical barriers often results in complications, prolonged hospital stays, and increased readmission rates, all of which drive up healthcare expenditures [16-18]. Additionally, patients with SDoH-related challenges are more likely to receive care in high-cost emergency settings than through preventive or outpatient services, further inflating costs [19,20].

While SDoH have been recognized as one of the key drivers of healthcare costs, their impact on hospitalization costs among orthopedic injury patients has not been directly examined using a nationally representative sample. Hospitalization costs are particularly important, as they comprise a substantial and well-characterized portion of healthcare expenditures associated with SDoH-related barriers. A large body of orthopedic trauma research has focused on financial and functional outcomes after injury, leaving a gap in understanding how SDoH may influence costs incurred during hospitalization. Examining this relationship is critical to understanding the economic burden of injury care. Considering these gaps in the literature, this study tests the hypothesis of whether SDoH contribute to variation in hospitalization costs among orthopedic injury patients, using a nationally representative dataset.

## Materials and methods

This study was not subject to Institutional Review Board (IRB) oversight since the data were provided in a de-identified format. We followed the Strengthening the Reporting of Observational Studies in Epidemiology (STROBE) guidelines to ensure comprehensive and transparent reporting of methods and findings.

Data source

We analyzed the 2016-2021 NIS, a weighted cross-sectional database, to estimate the national economic burden of fracture-related injuries among US adults [21]. The NIS is a discharge-level dataset that does not contain patient identifiers; therefore, repeat admissions for the same individual cannot be tracked. As the largest all-payer inpatient database in the US, NIS captures over 20% of hospitalizations and includes cost-to-charge ratio (CCR) data for deriving actual inpatient care costs. For this study, the CCR data were matched to the NIS using unique hospital identifiers, converting hospital charges into true expenditures, including wages, supplies, and utilities [22].

Study population

To identify patients with fracture-related injuries, we utilized the Clinical Classifications Software Refined (CCSR) for ICD-10-CM diagnoses [23]. For this study, we identified patients aged 18 years or older with initial or subsequent fracture-related injuries using the 12 CCSR diagnosis categories (INJ001-INJ006 and INJ038-INJ043) [24] for fractures of the head and neck, spine and back, torso, upper limb, lower limb (except hip), or neck of the femur (hip).

Outcome variable

The primary outcome variable in this study was the cost of hospitalization for inpatient admissions associated with fracture injuries. To allow comparison across years, total costs from each year (2016-2021) were adjusted to reflect 2024 US dollars. We applied year-specific inflation factors derived from the consumer price index (CPI) for medical care, published by the US Bureau of Labor Statistics [25]. For example, the cost from 2016 was multiplied by 1.29, meaning that $1 in 2016 is equivalent to $1.29 in 2024. This approach allowed us to account for inflation and make all cost estimates comparable over time.

SDoH categorization

The primary predictor of hospitalization costs was SDoH presence. Patients with SDoH-related diagnoses were identified using CCSR group FAC019, which includes 118 ICD-10 Z-codes documenting non-medical factors influencing health status and healthcare utilization [26]. All the ICD codes among these SDoH categories are provided in Appendix A.

Procedures, Elixhauser comorbidity and mortality score, and injury severity score (ISS)

Since fracture-related injuries may require significant therapeutic interventions, we utilized the CCSR for ICD-10-PCS procedures [27] to classify primary procedures as minor or major, diagnostic or therapeutic, and to identify operating room procedures.

Comorbidity and mortality scores were derived using the Elixhauser Comorbidity Software Refined for ICD-10-CM [28], which captures 38 pre-existing conditions. Injury severity was assessed using the ISS, generated through the ICD Program for Injury Categorization in R (ICDPIC-R) [29].

Statistical analysis

Demographic and clinical characteristics were summarized using descriptive statistics. Continuous variables were reported as medians (IQR) and categorical variables as frequencies (%). Group comparisons were performed using the Wilcoxon rank sum test for continuous variables and the Rao-Scott chi-square test for categorical variables. Analyses were based on complete case analysis; records with missing data on any covariates, the primary predictor (SDoH), or the outcome variable (hospitalization cost) were excluded.

Hospitalization costs were analyzed using multivariate linear regression stratified by SDoH status, adjusting for confounders. To ensure statistical validity, only mental, behavioral, and neurodevelopmental conditions with a prevalence greater than 1% were included. Due to the non-normal distribution of costs, a logarithmic transformation was applied. National total costs were estimated using the Healthcare Cost and Utilization Project (HCUP)-NIS weighting with survey procedures.

Temporal cost trends were modeled using ordinary least squares regression, with year as the independent variable. Future costs were projected using an exponential smoothing model (Proc ESM). All analyses were conducted in SAS 9.4 (SAS Institute Inc., Cary, NC), with statistical significance set at p<0.05.

## Results

Patient characteristics

**Figure 1:**
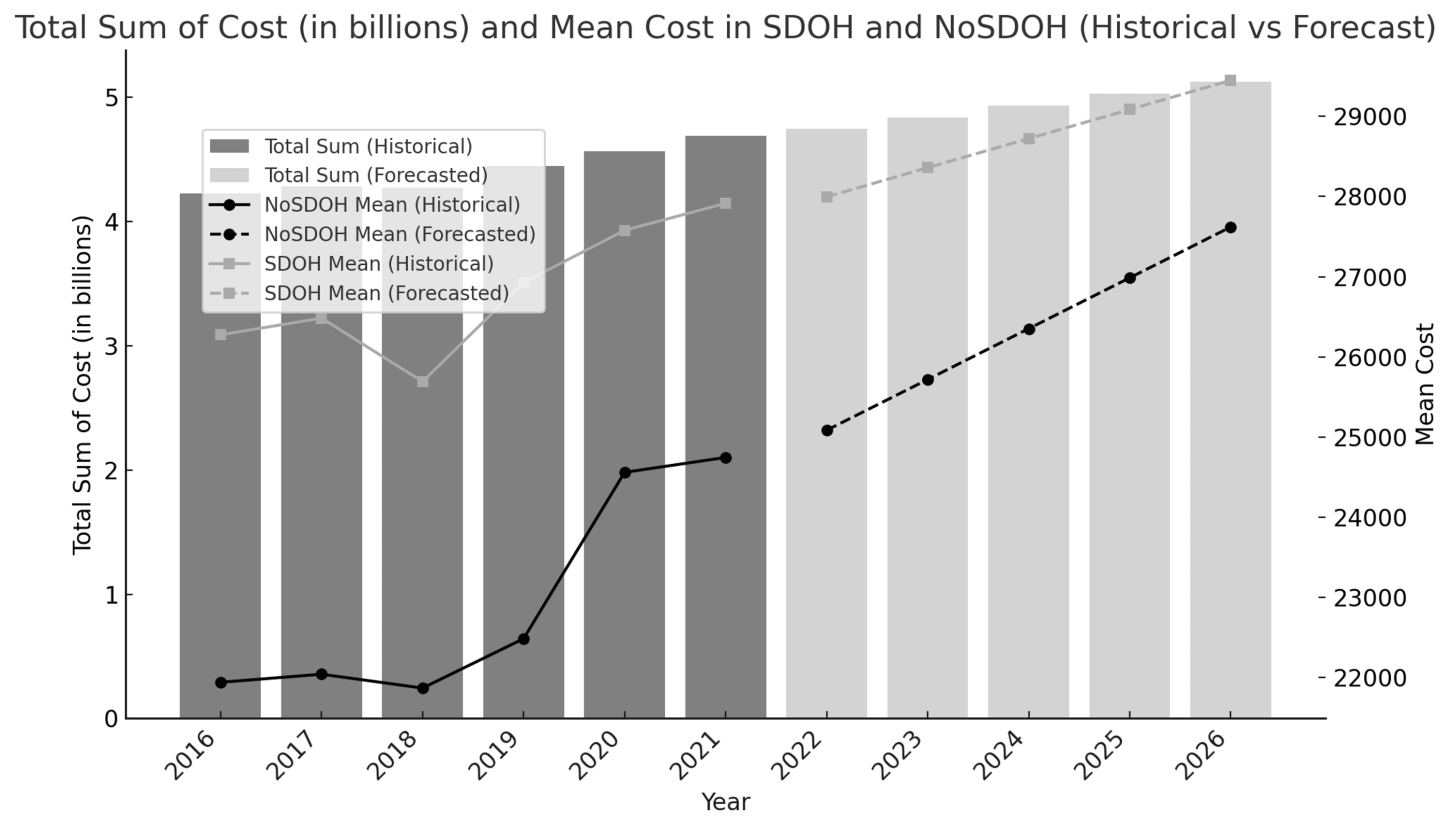
Total hospitalization cost (in billions) and mean cost per admission for orthopedic trauma patients with and without documented SDoH-related diagnoses (2016–2026; historical vs. forecasted) This figure presents trends in the total sum of hospitalization costs (bar graph, left y-axis) and mean cost per admission (line graph, right y-axis) for orthopedic trauma patients with and without documented social determinants of health (SDoH)-related diagnoses from 2016 to 2021 (historical) with forecasts through 2026. Dark gray bars represent the historical total sum of cost, and light gray bars represent forecasted values. Solid and dashed lines represent historical and forecasted mean costs for SDoH and NoSDoH groups, respectively.

We identified 1,160,566 orthopedic injury-related inpatient admissions, representing 5,802,827 hospitalizations. Patients with SDoH factors were younger (median: 58 years (IQR: 43-75) vs. median: 72 years (IQR: 57-84); p<0.001), of Black race (n=2,796 (14.02%) vs. n=89,160 (8.07%); p<0.001), and from the lowest income quartile (n=6,263 (36.23%) vs. n=313,161 (27.91%); p<0.001). Medicare was the predominant payer (n=713,848; 61.61%), but Medicaid coverage was higher in patients with SDoH (n=6,746 (32.90%) vs. n=102,284 (8.99%); p<0.001). Patients with SDoH factors had a higher 30-day readmission risk (median: 3 (IQR: 0-6) vs. median: 1 (IQR: 0-4); p<0.001). Patients with SDoH factors were more often hospitalized in urban teaching hospitals (n=16,183 (78.76%) vs. n= 813,546 (71.36%); p<0.001), had longer length of stay (LOS) (median: five days (IQR: 3-8) vs. median: four days (IQR 3-6); p<0.001), and more frequently had an emergency department record (n=17,219 (83.83%) vs. n=909,956 (79.82%); p<0.001). Mean hospitalization costs were higher in patients with SDoH (mean: $27,025.15 (SD: $31,052.87) vs. mean: $22,914.91 (SD: $25,155.04); p<0.001), contributing to an estimated $26.7 billion total cost. Total and mean hospitalization costs increased over time (Figure 1). Between 2016 and 2021, mean costs fluctuated, followed by a steady rise in projected estimates through 2026. The total sum of costs also increased, with forecasted values indicating continued growth in the coming years. Table 1 presents the patient and hospitalization characteristics.

**Table 1:** Patient and hospitalization characteristics for fracture-related orthopedic trauma admissions by the presence of documented SDoH-related diagnoses (unweighted n=1,160,566) SDoH: Social determinants of health; NIS: National inpatient sample; ED: Emergency department; ISS: Injury severity score; IQR: Interquartile range; SD: Standard deviation; ICD-10: International Classification of Diseases, 10th Revision. All costs are inflation-adjusted to 2024 USD using the consumer price index. Statistical comparisons used Rao–Scott chi-square tests for categorical variables, and Wilcoxon two-sample or t-tests for continuous variables, as appropriate. Cell values less than 10, as well as any smaller subsequent values, have been masked in accordance with the NIS data use guidelines. P-values <0.05 were considered statistically significant. ** Cell values less than 10, as well as any smaller subsequent values, have been masked.

Characteristics	Overall	NoSDoH	SDoH	p-value	Test statistics	Test name
	n (%)	n (%)	n (%)			
	1,160,566 (100)	1,140,019 (98.23)	20,547 (1.77)			
2016	194,900 (16.79)	192,482 (16.88)	2418 (11.77)	-		
2017	194,507 (16.76)	192,039 (16.85)	2468 (12.01)			
2018	195,457 (16.84)	192,716 (16.90)	2741 (13.34)			
2019	198,126 (17.07)	194,721 (17.08)	3405 (16.57)			
2020	187,027 (16.12)	182,995 (16.05)	4032 (19.62)			
2021	190,549 (16.42)	185,066 (16.23)	5483 (26.69)			
Age at admission, Median (IQR)	72 (56-84)	72 (57-84)	58 (43-75)	<0.0001	-67.80	Wilcoxon Two-Sample Test (z-statistics)
Sex, Female	680,208 (58.62)	671,644 (58.93)	8564 (41.69)	<0.0001	1440.18	Rao-Scott Chi-Square
Race						
White	880,643 (78.25)	866,854 (78.42)	13,789 (69.12)	<0.0001	786.82	Rao-Scott Chi-Square
Black	880,643 (78.25)	89,160 (8.07)	2796 (14.02)			
Hispanic	96,490 (8.57)	94,313 (8.53)	2177 (10.91)			
Asian or Pacific Islander	21,261 (1.89)	20,952 (1.90)	309 (1.55)			
Native American	6508 (0.58)	6232 (0.56)	276 (1.38)			
Other/Unknown	28,554 (2.54)	27,953 (2.53)	601 (3.01)			
Missing	35,154	34,555	599			
Patient Income						
0-25th percentile	319,424 (28.04)	313,161 (27.91)	6263 (36.23)	<0.0001	334.90	Rao-Scott Chi-Square
26th-50th percentile (median)	303,448 (26.64)	298,909 (26.64)	4539 (26.26)			
51st-75th percentile	303,448 (26.64)	273,546 (24.38)	3761 (21.76)			
76th-100th percentile	238,964 (20.98)	236,241 (21.06)	2723 (15.75)			
Missing	21,423	18,162	3261			
Patient Insurance Type						
Medicare	713,848 (61.61)	705,481 (61.99)	8367 (40.80)	<0.0001	7172.73	Rao-Scott Chi-Square
Medicaid	109,030 (9.41)	102,284 (8.99)	6746 (32.90)			
Private insurance	226,123 (19.52)	223,925 (19.68)	2198 (10.72)			
Self-pay	51,014 (4.40)	49,120 (4.32)	1894 (9.24)			
No charge	4211 (0.36)	3953 (0.35)	258 (1.26)			
Other	54,365 (4.69)	53,321 (4.69)	1044 (5.09)			
Missing	1975	1935	40			
Patient Location						
Metro	586,936 (50.57)	577,531 (50.66)	9405 (45.77)	<0.0001	165.65	Rao-Scott Chi-Square
Urban	360,527 (31.06)	354,700 (31.11)	5827 (28.36)			
Micropolitan/Rural	213,103 (18.36)	207,788 (18.23)	5315 (25.87)			
Hospital Location						
Rural	105,244 (9.07)	104,130 (9.13)	1114 (5.42)	<0.0001	168.20	Rao-Scott Chi-Square
Urban nonteaching	105,244 (9.07)	222,343 (19.50)	3250 (15.82)			
Urban teaching	829,729 (71.49)	813,546 (71.36)	16,183 (78.76)			
Length of Stay, Median (IQR)	4 (3-7)	4 (3-6)	5 (3-8)	<0.0001	26.37	Wilcoxon Two-Sample Test (z-statistics)
Total Number of Procedures, Median (IQR)	1 (1-2)	1 (1-2)	1 (0-3)	<0.0001	2.93	Wilcoxon Two-Sample Test (z-statistics)
Primary Listed Procedure Class						
Minor Diagnostic	17,288 (1.97)	16,897 (1.96)	391 (2.64)	<0.0001	258.80	Rao-Scott Chi-Square
Minor Therapeutic	123,665 (14.07)	120,858 (13.99)	2807 (18.93)			
Major Diagnostic	1253 (0.14)	1232 (0.14)	21 (0.14)			
Major Therapeutic	736,702 (83.82)	725,094 (83.92)	11,608 (78.29)			
Missing/unknown	281,658	275,938	5720			
Operating room procedure reported on the record	755,204 (65.07)	743,258 (65.20)	11,946 (58.14)	<0.0001	298.81	Rao-Scott Chi-Square
Indication of Emergency Department Record	927,175 (79.89)	909,956 (79.82)	17,219 (83.83)	<0.0001	61.82	Rao-Scott Chi-Square
Cost (2024 USD)						
Mean (SD)	$22,987.04 ($25,276.15)	$22,914.91 ($25,155.04)	$27,025.15 ($31,052.87)	<0.0001	-18.71	Two Sample T-Test (t-value)
Median (IQR)	$17,571 ($11,125-$26,513)	$17,547 ($11,118-$26,437)	$19,233 ($11,567-$31,239)	<0.0001	19.58	Wilcoxon Two-Sample Test (z-statistics)
Total Elixhauser Comorbid Conditions, Median (IQR)	2 (1-3)	2 (1-3)	2 (1-3)	<0.0001	-4.99	Wilcoxon Two-Sample Test (z-statistics)
30-day, all-cause readmission, Median (IQR)	1 (0-4)	1 (0-4)	3 (0-6)	<0.0001	45.82	Wilcoxon Two-Sample Test (z-statistics)
Risk of in-hospital mortality, Median (IQR)	0 (-3 to 1)	0 (-3 to 1)	-1 (-7 to 0)	<0.0001	-49.38	Wilcoxon Two-Sample Test (z-statistics)
Injury Severity Score (ISS), Median (IQR)	4 (1-9)	4 (1-9)	4 (1-9)	0.0002	-8.55	Wilcoxon Two-Sample Test (z-statistics)
Fracture Location						
Head and neck, initial encounter	72,667 (6.26)	69,643 (6.11)	3024 (14.72)	<0.0001	1684.62	Rao-Scott Chi-Square
Spine and back, initial encounter	184,589 (15.91)	181,220 (15.90)	3369 (16.40)	0.0967	2.76	Rao-Scott Chi-Square
Torso, initial encounter	208,077 (17.93)	203,897 (17.89)	4180 (20.34)	<0.0001	58.77	Rao-Scott Chi-Square
Upper limb, initial encounter	192,638 (16.60)	188,796 (16.56)	3842 (18.70)	<0.0001	56.12	Rao-Scott Chi-Square
Lower limb (except hip), initial encounter	283,930 (24.46)	278,122 (24.40)	5808 (28.27)	<0.0001	130.36	Rao-Scott Chi-Square
Neck of the femur (hip), initial encounter	403,912 (34.80)	399,088 (35.01)	4824 (23.48)	<0.0001	815.81	Rao-Scott Chi-Square
Head and neck, subsequent encounter	3018 (0.26)	2903 (0.25)	115 (0.56)	<0.0001	62.51	Rao-Scott Chi-Square
Spine and back, subsequent encounter	16,980 (1.46)	16,655 (1.46)	325 (1.58)	0.1898	1.72	Rao-Scott Chi-Square
Torso, subsequent encounter	17,471 (1.51)	17,116 (1.50)	355 (1.73)	0.02	5.41	Rao-Scott Chi-Square
Upper limb, subsequent encounter	17,971 (1.55)	17,614 (1.55)	357 (1.74)	0.0341	4.49	Rao-Scott Chi-Square
Lower limb (except hip), subsequent encounter	27,495 (2.37)	26,990 (2.37)	505 (2.46)	0.4735	0.51	Rao-Scott Chi-Square
Neck of the femur (hip), subsequent encounter	35,762 (3.08)	35,183 (3.09)	579 (2.82)	0.1205	2.41	Rao-Scott Chi-Square
Intent of Injury						
Assault	20,210 (1.74)	18,205 (1.60)	2005 (9.76)	<0.0001	4256.83	Rao-Scott Chi-Square
Self-inflicted	1731 (0.15)	1566 (0.14)	165 (0.80)			
Unintentional	858,256 (73.95)	84,3795 (74.02)	14,461 (70.38)			
Not Available/Other/Undetermined	280,369 (24.16)	276,453 (24.25)	3916 (19.06)			
Mechanism of Injury						
Adverse effects	5529 (0.48)	5456 (0.48)	73 (0.36)	<0.0001	6893.21	Chi-square Statistics Fisher's Exact
Cut-pierce	2006 (0.17)	1919 (0.17)	87 (0.42)			
Drowning	37 (0.00)	**	**			
Fall	611,847 (52.72)	603,083 (52.90)	8764 (42.65)			
Fire/Burn	425 (0.04)	413 (0.04)	12 (0.06)			
Firearm	11,803 (1.02)	11,319 (0.99)	484 (2.36)			
MVC	112,051 (9.65)	109,130 (9.57)	2921 (14.22)			
Machinery	2921 (0.25)	2904 (0.25)	17 (0.08)			
Natural/Environmental	2629 (0.23)	2581 (0.23)	48 (0.23)			
Overexertion	5475 (0.47)	5391 (0.47)	84 (0.41)			
Pedal cyclist	8923 (0.77)	8577 (0.75)	346 (1.68)			
Pedestrian	2769 (0.24)	2599 (0.23)	170 (0.83)			
Poisoning	55 (0.00)	**	**			
Struck by, against	18,638 (1.61)	17,234 (1.51)	1404 (6.83)			
Transport	79,542 (6.85)	78,021 (6.84)	1521 (7.40)			
Other	24,734 (2.13)	24,056 (2.11)	678 (3.30)			
Not Available	269,195 (23.20)	265,486 (23.29)	3709 (18.05)			
Unspecified	1987 (0.17)	1758 (0.15)	229 (1.11)			

Hip fractures were most common (n=403,912; 34.80%) but less frequent in patients with SDoH (n=4,824 (23.48%) vs. n=399,088 (35.01%); p<0.001). Patients with SDoH factors had more fractures in the lower limb, excluding hip (n=5,808 (28.27%) vs. n=278,122 (24.40%); p<0.001), torso (n=4,180 (20.34%) vs. n=203,897 (17.89%); p<0.001), and upper limb (n=3,842 (18.70%) vs. n=188,796 (16.56%); p<0.001). Assault-related (n=2,005 (9.76%) vs. n=18,205 (1.60%); p<0.001) were more common in patients with SDoH factors. Falls were the leading mechanism (n=611,847; 52.72%), but MVC-related (n=2,921 (14.22%) vs. n=109,130 (9.57%); p<0.001) and firearm-related injuries (n=484 (2.36%) vs. n=11,319 (0.99%); p<0.001) were more frequent in patients with SDoH factors.

Comorbid conditions

Comorbidities differed significantly by SDoH status. Patients with SDoH factors had higher rates of alcohol (n=4,930 (23.99%) vs. n=75,914 (6.66%); p<0.001) and drug abuse (n=3,868 (18.83%) vs. n=33,953 (2.98%); p<0.001). From CCSR categorization of mental health disorders, among patients with SDoH factors disorders, including schizophrenia (n=1,688 (8.22%) vs. n=15,042 (1.32%); p<0.001) and bipolar disorder (n=1,356 (6.60%) vs. n=21,571 (1.89%); p<0.001), were more common. Substance use disorders were also more prevalent in patients with SDoH factors, particularly alcohol-related (n=4,909 (23.89%) vs. n=76,614 (6.72%); p<0.001) and opioid-related disorders (n=1,589 (7.73%) vs. n=33,813 (2.97%); p<0.001). Suicidal ideation or intentional self-harm was also significantly higher (n=707 (3.44%) vs. n=4,401 (0.39%); p<0.001) in patients with SDoH. Table 2 presents the Elixhauser comorbid conditions and mental or behavioral health conditions among orthopedic trauma admissions.

**Table 2:** Elixhauser comorbid conditions and mental or behavioral health conditions among orthopedic trauma admissions by presence of documented SDoH-related diagnoses (unweighted n=1,160,566) SDoH: Social determinants of health; CCSR: Clinical Classifications Software Refined; ICD-10: International Classification of Diseases, 10th Revision; NIS: National Inpatient Sample All comparisons were conducted using Rao–Scott chi-square tests. P-values <0.05 were considered statistically significant. Cell values less than 10, as well as any smaller subsequent values, have been masked in accordance with the NIS data use guidelines. ** Cell values less than 10, as well as any smaller subsequent values, have been masked.

Characteristics	Overall	NoSDoH	SDoH	p-value	Test statistics	Test name
	n (%)	n (%)	n (%)			
	1,160,566 (100)	1,140,019 (98.23)	20547 (1.77)			
Elixhauser Comorbid Conditions						
AIDS - Acquired immune deficiency syndrome	3939 (0.34)	3671 (0.32)	268 (1.30)	<0.0001	550.40	Rao-Scott Chi-Square
Alcohol abuse	80,844 (6.97)	75,914 (6.66)	4930 (23.99)	<0.0001	6625.31	Rao-Scott Chi-Square
Autoimmune conditions	43,850 (3.78)	43,388 (3.81)	462 (2.25)	<0.0001	126.52	Rao-Scott Chi-Square
Lymphoma	6317 (0.54)	6263 (0.55)	54 (0.26)	<0.0001	30.87	Rao-Scott Chi-Square
Leukemia	5223 (0.45)	5173 (0.45)	50 (0.24)	<0.0001	19.60	Rao-Scott Chi-Square
Metastatic cancer	9649 (0.83)	9555 (0.84)	94 (0.46)	<0.0001	34.79	Rao-Scott Chi-Square
Solid tumor without metastasis, in situ	200 (0.02)	**	**	0.4082	0.68	Rao-Scott Chi-Square
Solid tumor without metastasis, malignant	19,386 (1.67)	19,135 (1.68)	251 (1.22)	<0.0001	24.14	Rao-Scott Chi-Square
Cerebrovascular disease	28,436 (2.45)	28,074 (2.46)	362 (1.76)	<0.0001	40.77	Rao-Scott Chi-Square
Heart failure	178 (0.02)	178 (0.02)	0	-		
Dementia	169,996 (14.65)	168,632 (14.79)	1364 (6.64)	<0.0001	881.27	Rao-Scott Chi-Square
Depression	166,876 (14.38)	163,548 (14.35)	3328 (16.20)	<0.0001	42.96	Rao-Scott Chi-Square
Diabetes without chronic complications	115,067 (9.91)	113,655 (9.97)	1412 (6.87)	<0.0001	212.82	Rao-Scott Chi-Square
Diabetes with chronic complications	138,509 (11.93)	136,712 (11.99)	1797 (8.75)	<0.0001	177.97	Rao-Scott Chi-Square
Drug abuse	37,821 (3.26)	33,953 (2.98)	3868 (18.83)	<0.0001	13,608.81	Rao-Scott Chi-Square
Hypertension, complicated	223,306 (19.24)	220,829 (19.37)	2477 (12.06)	<0.0001	460.20	Rao-Scott Chi-Square
Hypertension, uncomplicated	480,740 (41.42)	473,245 (41.51)	7495 (36.48)	<0.0001	159.38	Rao-Scott Chi-Square
Liver disease, moderate to severe	1176 (0.10)	**	**	0.0089	6.84	Rao-Scott Chi-Square
Chronic pulmonary disease	215,596 (18.58)	211,669 (18.57)	3927 (19.11)	0.0548	3.69	Rao-Scott Chi-Square
Obesity	120,663 (10.40)	118,898 (10.43)	1765 (8.59)	<0.0001	48.63	Rao-Scott Chi-Square
Paralysis	22,165 (1.91)	21,878 (1.92)	287 (1.40)	<0.0001	29.50	Rao-Scott Chi-Square
Peripheral vascular disease	64,317 (5.54)	63,442 (5.56)	875 (4.26)	<0.0001	60.94	Rao-Scott Chi-Square
Renal failure, severe	18,165 (1.57)	18,009 (1.58)	156 (0.76)	<0.0001	85.84	Rao-Scott Chi-Square
Hypothyroidism	19,4276 (16.74)	192,151 (16.86)	2125 (10.34)	<0.0001	457.06	Rao-Scott Chi-Square
Other thyroid disorders	14,354 (1.24)	14,117 (1.24)	237 (1.15)	0.2902	1.12	Rao-Scott Chi-Square
Valvular disease	18,295 (1.58)	18,101 (1.59)	194 (0.94)	<0.0001	49.43	Rao-Scott Chi-Square
Mental, behavioral and neurodevelopmental disorders (from CCSR)						
Schizophrenia spectrum and other psychotic disorders	16,730 (1.44)	15,042 (1.32)	1688 (8.22)	<0.0001	5713.49	Rao-Scott Chi-Square
Depressive disorders	167,801 (14.46)	164,459 (14.43)	3342 (16.27)	<0.0001	42.20	Rao-Scott Chi-Square
Bipolar and related disorders	22,927 (1.98)	21,571 (1.89)	1356 (6.60)	<0.0001	2158.29	Rao-Scott Chi-Square
Other specified and unspecified mood disorders	4561 (0.39)	4373 (0.38)	188 (0.91)	<0.0001	138.74	Rao-Scott Chi-Square
Anxiety and fear-related disorders	158,613 (13.67)	155,201 (13.61)	3412 (16.61)	<0.0001	126.36	Rao-Scott Chi-Square
Obsessive-compulsive and related disorders	1727 (0.15)	1685 (0.15)	42 (0.20)	0.0408	4.18	Rao-Scott Chi-Square
Trauma- and stressor-related disorders	18,326 (1.58)	17,217 (1.51)	1109 (5.40)	<0.0001	1757.28	Rao-Scott Chi-Square
Disruptive, impulse-control and conduct disorders	893 (0.08)	764 (0.07)	129 (0.63)	<0.0001	808.24	Rao-Scott Chi-Square
Personality disorders	2230 (0.19)	1948 (0.17)	282 (1.37)	<0.0001	1149.11	Rao-Scott Chi-Square
Feeding and eating disorders	389 (0.03)	376 (0.03)	13 (0.06)	0.0191	5.49	Rao-Scott Chi-Square
Somatic disorders	447 (0.04)	428 (0.04)	19 (0.09)	0.0002	14.25	Rao-Scott Chi-Square
Suicidal ideation/attempt/intentional self-harm	5108 (0.44)	4401 (0.39)	707 (3.44)	<0.0001	3953.84	Rao-Scott Chi-Square
Miscellaneous mental and behavioral disorders/conditions	1996 (0.17)	1904 (0.17)	92 (0.45)	<0.0001	94.74	Rao-Scott Chi-Square
Neurodevelopmental disorders	12,497 (1.08)	12,091 (1.06)	406 (1.98)	<0.0001	161.48	Rao-Scott Chi-Square
Alcohol-related disorders	81,523 (7.02)	76,614 (6.72)	4909 (23.89)	<0.0001	6573.56	Rao-Scott Chi-Square
Opioid-related disorders	35,402 (3.05)	33,813 (2.97)	1589 (7.73)	<0.0001	1190.68	Rao-Scott Chi-Square
Cannabis-related disorders	24,584 (2.12)	22,772 (2.00)	1812 (8.82)	<0.0001	4249.36	Rao-Scott Chi-Square
Sedative-related disorders	3698 (0.32)	3512 (0.31)	186 (0.91)	<0.0001	223.90	Rao-Scott Chi-Square
Stimulant-related disorders	17,777 (1.53)	14,801 (1.30)	2976 (14.48)	<0.0001	21,450.16	Rao-Scott Chi-Square
Hallucinogen-related disorders	632 (0.05)	528 (0.05)	104 (0.51)	<0.0001	699.23	Rao-Scott Chi-Square
Inhalant-related disorders	209 (0.02)	**	**	0.8755	0.02	Rao-Scott Chi-Square
Tobacco-related disorders	185,313 (15.97)	177,228 (15.55)	8085 (39.35)	<0.0001	5710.90	Rao-Scott Chi-Square
Other specified substance-related disorders	5374 (0.46)	4771 (0.42)	603 (2.93)	<0.0001	2569.68	Rao-Scott Chi-Square
Mental and substance use disorders in remission	8413 (0.72)	8107 (0.71)	306 (1.49)	<0.0001	160.03	Rao-Scott Chi-Square
Suicide attempt/intentional self-harm; subsequent encounter	95 (0.01)	**	**	<0.0001	11.54	Rao-Scott Chi-Square
Opioid-related disorders; subsequent encounter	364 (0.03)	**	**	0.078	3.11	Rao-Scott Chi-Square

Predictors of hospitalization cost

Each additional hospitalization day increased costs (NoSDoH: 4.0%, p<0.0001; SDoH: 3.1%, p<0.0001), as did each procedure (NoSDoH: 14.0%, p<0.0001; SDoH: 13.2%, p<0.0001). Rural hospitalizations raised costs in NoSDoH (3.3%, p<0.0001) but lowered them in SDoH (-17.1%, p<0.0001). Urban non-teaching hospitals had lower costs (NoSDoH: -2.1%, p=0.0001; SDoH: -3.9%, p=0.0206). Urban patient residence reduced costs in patients without SDoH factors (-4.8%, p<0.0001). Micropolitan/rural areas had lower costs in NoSDoH (-4.5%, p<0.0001) but higher costs in patients with SDoH factors (18.0%, p<0.0001). Medicaid (NoSDoH: 4.5%, p<0.0001; SDoH: 11.7%, p<0.0001) and private insurance (NoSDoH: 4.7%, p<0.0001; SDoH: 8.4%, p<0.0001) increased costs. Self-pay (NoSDoH: -4.4%, p<0.0001; SDoH: -7.8%, p<0.0001) and "No charge" status (NoSDoH: -7.6%, p<0.0001; SDoH: -13.7%, p<0.0001) lowered costs. Table 3 presents the exponentiated multivariate regression results showing the percent change in hospitalization costs.

**Table 3:** Exponentiated multivariate regression results showing percent change in hospitalization costs, stratified by the SDoH status SDoH: Social Determinants of Health; CI: Confidence interval Estimate values represent exponentiated regression coefficients from multivariable linear regression models. These reflect the percent change in hospitalization costs associated with each predictor, holding all other variables constant. Separate models were fit for patients with and without documented SDoH-related diagnoses. The intercept corresponds to the estimated average cost of hospitalization when all predictors are set to zero. P-values <0.05 were considered statistically significant. *Represents the exponentiated regression coefficient obtained form the multivariate regression analysis. These values represent the difference in costs between categories and per unit change. Among the NoSDOH samples, for example, the difference of cost between opioid-related disorder compared to no such disorder is 7% (exp: 0.0686). The intercept is interpreted as at the average cost of hospitalization is 10,728 (exp: 9.281) when all the other variables are at zero.

Parameter		NoSDoH					SDoH				
		(n=1130096)					(n=20180)				
		Estimate*	95% CI		p-value	t-value	Estimate*	95% CI		p-value	t-value
Length of Stay		1.040	1.039	1.041	<0.0001	60.04	1.031	1.028	1.034	<0.0001	19.10
Readmission Risk		1.005	1.005	1.005	<0.0001	22.38	1.003	1.000	1.005	0.051	1.95
Number of Procedures		1.140	1.138	1.143	<0.0001	111.23	1.132	1.125	1.139	<0.0001	39.12
Hospital Location	Rural vs Urban Teaching	1.033	1.018	1.050	<0.0001	4.18	0.829	0.784	0.877	<0.0001	-6.53
	Urban Non-teaching vs Urban Teaching	0.979	0.968	0.989	0.0010	-3.84	0.961	0.929	0.994	0.021	-2.32
Patient Location	Urban vs Metro	0.952	0.943	0.962	<0.0001	-9.48	0.976	0.950	1.003	0.085	-1.72
	Micropolitan/Rural vs Metro	0.955	0.943	0.967	<0.0001	-7.27	1.180	1.138	1.224	<0.0001	8.91
Insurance Status	Medicaid vs Medicare	1.045	1.035	1.054	<0.0001	9.42	1.117	1.087	1.146	<0.0001	8.17
	Private insurance vs Medicare	1.047	1.042	1.053	<0.0001	17.41	1.084	1.048	1.121	<0.0001	4.70
	Self-pay vs Medicare	0.956	0.944	0.968	<0.0001	-7.21	0.922	0.890	0.955	<0.0001	-4.48
	No charge vs Medicare	0.924	0.896	0.954	<0.0001	-4.97	0.863	0.801	0.929	<0.0001	-3.91
	Other vs Medicare	1.012	1.002	1.022	0.021	2.30	0.974	0.913	1.040	0.433	-0.79
Schizophrenia spectrum and other psychotic disorders	Yes vs No	1.006	0.995	1.017	0.284	1.07	1.035	1.001	1.070	0.044	2.01
Depressive disorders	Yes vs No	0.989	0.986	0.993	<0.0001	-5.49	0.976	0.951	1.001	0.062	-1.87
Bipolar and related disorders	Yes vs No	0.991	0.983	1.000	0.043	-2.02	0.967	0.931	1.005	0.086	-1.72
Anxiety and fear-related disorders	Yes vs No	0.998	0.994	1.001	0.228	-1.21	1.009	0.984	1.034	0.497	0.68
Trauma- and stressor-related disorders	Yes vs No	0.962	0.949	0.975	<0.0001	-5.63	1.009	0.968	1.053	0.669	0.43
Neurodevelopmental disorders	Yes vs No	1.024	1.013	1.036	<0.0001	4.23	1.016	0.952	1.084	0.635	0.47
Alcohol-related disorders	Yes vs No	1.032	1.027	1.038	<0.0001	11.69	0.989	0.967	1.012	0.347	-0.94
Opioid-related disorders	Yes vs No	1.071	1.064	1.078	<0.0001	19.51	1.060	1.024	1.097	0.001	3.30
Cannabis-related disorders	Yes vs No	1.044	1.033	1.054	<0.0001	8.06	1.025	0.993	1.059	0.127	1.53
Stimulant-related disorders	Yes vs No	1.062	1.048	1.076	<0.0001	8.95	1.095	1.063	1.129	0.0001	5.89
Tobacco-related disorders	Yes vs No	1.003	0.999	1.007	0.195	1.30	0.984	0.964	1.004	0.116	-1.57
Intercept		10,728.027	10,624.685	10,832.374	<0.0001	1879.29	11,056.116	10,726.732	11,395.614	<0.0001	603.50

Depressive (-1.1%, p<0.0001) and bipolar disorders (-0.9%, p=0.043) reduced costs in patients without SDoH. Schizophrenia spectrum disorders had no impact on patients without SDoH (p=0.284) but increased costs in patients with SDoH factors(3.5%, p=0.044). Opioid-related (NoSDoH: 7.1%, p<0.0001; SDoH: 6.0%, p=0.001) and stimulant-related disorders (NoSDoH: 6.2%, p<0.0001; SDoH: 9.5%, p<0.0001) increased costs in both groups. Cannabis-related (4.4%, p<0.0001) and alcohol-related (3.2%, p<0.0001) disorders increased costs in patients without SDoH but were not significant in patients with SDoH factors (p=0.1267; p=0.347, respectively).

## Discussion

This study provides critical insights into the impact of SDoH on hospitalization costs among patients with fracture-related orthopedic injuries, utilizing a nationally representative dataset. Our findings underscore the significant financial and healthcare disparities associated with SDoH factors, highlighting their role as key drivers of increased hospitalization costs and disparities in access, treatment, and outcomes.

Patients with SDoH factors incurred hospitalization costs 18% higher than their counterparts based on unadjusted mean differences reported in Table 1, reflecting the financial burden of caring for vulnerable populations. These elevated costs stem from delayed or fragmented care, leading to more severe injuries, prolonged hospital stays, and greater resource utilization. Research has linked lower socioeconomic status with more advanced injury presentations and treatment at low-volume facilities, further increasing healthcare expenditures [30]. These findings highlight systemic barriers, including inadequate insurance and limited preventive care access, that exacerbate disparities in orthopedic outcomes.

Demographic patterns associated with SDoH factors were particularly pronounced. Patients with SDoH factors were typically younger, more likely to belong to minority racial and ethnic groups, and disproportionately represented in the lowest income quartile, indicating that socioeconomic and racial inequities contributed to injury risk and healthcare needs [31,32]. Prior research found that lower educational attainment and income levels were associated with a higher likelihood of traumatic brain injury, with minority populations experiencing disproportionately higher risks than their white counterparts [33]. Additionally, as corroborated by previous research, these patients were more likely to reside in rural areas with limited healthcare access, had higher rates of emergency department admissions, and received care at urban teaching hospitals, which often served as safety nets for underserved populations [3,34-36].

Rural healthcare access challenges are well-documented, with provider shortages, limited specialty care availability, and financial constraints contributing to disparities in both the timeliness and quality of orthopedic care [37]. Many rural patients delay seeking medical attention due to a tendency to avoid doctor visits, postpone care because of cost, or refrain from disclosing illness [38], potentially leading to more severe injury presentations, prolonged hospital stays, and increased healthcare costs. Rural patients also report higher satisfaction in safety-net hospitals compared to non-safety-net hospitals [39] trend observed in the current study. Another study corroborated these findings, noting that greater rurality was associated with decreased patient satisfaction, primarily due to travel distance and limited availability of specialists [38]. Furthermore, transportation barriers exacerbate healthcare disparities, as rural patients often travel long distances to receive specialty care, increasing the likelihood of delayed or missed follow-up appointments and suboptimal recovery outcomes [40].

Given these challenges, targeted interventions are essential to mitigating disparities. Expanding telemedicine for orthopedic consultations and follow-up appointments can reduce travel burdens and increase specialist access [35]. Increasing financial support for rural hospitals, including increasing Medicaid reimbursements and loan forgiveness for rural physicians, may alleviate provider shortages and enhance access to care [41]. Community-based health education initiatives can further address healthcare hesitancy by promoting early intervention, improving health literacy, and encouraging adherence to treatment plans [42,43].

The study identified significant disparities in injury mechanisms and affected body regions, consistent with findings from previous research. As observed in prior literature, patients with SDoH factors were disproportionately more likely to sustain firearm- and assault-related injuries may involve intimate partner violence [44-46], while falls remained the predominant cause of injury across all groups. However, this study provides additional insights, demonstrating that patients without SDoH factors had a higher prevalence of hip fractures, whereas those with SDoH factors experienced more fractures in the limbs and torso. These differences may reflect variations in environmental hazards, occupational risks, and disparities in housing conditions [47,48], which contribute to the increased likelihood of specific injury types among socioeconomically disadvantaged populations.

Among patients in the SDoH group, substance use disorders, including opioid and stimulant-related conditions, were notably prevalent, as documented in previous research [49]. These disorders are associated with increased healthcare resource utilization, including extended hospital stays [50] and prolonged medical management [51,52]. In the present study, substance use disorders were also a significant driver of healthcare costs among patients with and without SDoH factors, further exacerbating financial disparities in trauma care. Given the well-established link between substance use and injury risk, implementing a multi-agency approach such as community-based discharge planning [53] and transitional discharge model [54] may improve patient outcomes, reduce LOS, and optimize healthcare expenditures.

The observed differences in payer types and their impact on hospitalization costs highlight systemic inefficiencies in healthcare financing and access. While Medicare remained the predominant payer overall, Medicaid coverage was associated with higher hospitalization costs across all patients, with the financial burden being even greater among those with SDoH factors. This trend underscores the structural challenges within public insurance programs, where Medicaid beneficiaries, both with and without SDoH factors, face systemic barriers leading to greater reliance on inpatient care, prolonged hospitalizations, and higher resource utilization. These cost disparities likely stem from limited access to preventive services, fragmented care coordination, and the higher prevalence of comorbid conditions in low-income populations [17].

From a health policy perspective, these findings reinforce the need for Medicaid delivery system reforms to address inefficiencies that contribute to preventable hospitalizations and escalating costs. Medicaid expansion under the Affordable Care Act has improved healthcare access and reduced uncompensated care costs, but gaps remain in addressing the social drivers of high-cost hospitalizations, particularly for those with SDoH factors. Policymakers must consider integrating social services within Medicaid programs, as recommended by the Centers for Medicare & Medicaid Services, through initiatives such as the Accountable Health Communities Model and Medicaid Section 1115 Demonstration Waivers to improve care coordination and reduce avoidable hospitalizations [55]. These programs allow states to pilot integrated care models that reimburse for housing support, transportation, and other nonmedical services that influence health outcomes. Systemic reforms should shift Medicaid spending from high-cost inpatient services to community-based care and prevention. Value-based payment models, such as CMS’s Medicaid Integrated Care for Kids (InCK) Model and State Innovation Models (SIM), further encourage healthcare systems to address social risks by tying reimbursement to quality and cost metrics rather than volume of care. Expanding these models and increasing reimbursement for social risk interventions, such as care coordination and community health worker programs, could reduce preventable hospitalizations.

This study has several limitations. First, propensity score matching was not used, as SDoH-related disparities encompass complex, interdependent factors that cannot be fully accounted for through statistical matching alone. Alternative approaches, such as causal inference methods or machine learning-based risk stratification, may provide a more comprehensive assessment. Second, race and sex were excluded from the multivariate analysis due to their high collinearity with key SDoH variables, such as income, insurance status, and geographic location. Furthermore, race and sex are unmodifiable factors and may function as proxies for broader structural inequities rather than direct determinants of healthcare costs. Adjusting for these variables without fully accounting for systemic disparities such as differential access to care and implicit bias in treatment could introduce residual confounding and lead to biased estimates. Third, reliance on administrative data limits the ability to assess patient-reported social risks, functional outcomes, and long-term recovery trajectories. Future research should incorporate longitudinal datasets, qualitative methodologies, and patient-centered measures to provide a comprehensive understanding of how SDoH influences orthopedic trauma care beyond the hospitalization period. Finally, the underreporting of SDoH diagnoses remains a significant limitation, likely leading to an underestimation of the true financial and clinical burden associated with these factors.

Despite its limitations, by leveraging a nationally representative database, this study offers a comprehensive assessment of the relationship between SDoH and healthcare expenditures, ensuring the generalizability of findings across diverse patient populations. One of the key strengths of this study is its ability to capture trends in SDoH diagnosis coding. Prior to 2018, only clinicians were authorized to document SDoH-related diagnoses [26]; however, subsequent changes in reporting guidelines expanded this capability to include additional healthcare providers. This study reflects that shift, demonstrating an increase in documented SDoH-related diagnoses over time. However, we recognize that administrative coding of SDoH may not fully capture the complexity of underlying social, racial, and economic disparities. SDoH codes reflect documentation practices rather than the presence or severity of social risk itself.

Nevertheless, the underreporting of SDoH in orthopedic literature remains a significant issue, with only 10% of studies incorporating SDoH factors such as race, ethnicity, employment status, insurance status, and education level [30,56]. This limitation highlights the challenges of relying on administrative data to measure subtle social conditions and underscores the need to distinguish coded SDoH variables from broader systemic inequities. Emerging strategies to improve documentation include integrating standardized tools such as PRAPARE into electronic health records (EHRs) [57], which have demonstrated feasibility and impact in safety net and primary care settings [58,59]. National initiatives such as the Gravity Project are also working to standardize SDoH terminology across systems, enabling more consistent and actionable coding [60].

These efforts, particularly when coupled with value-based payment incentives, can enhance the accuracy and utility of SDoH data and lay the groundwork for more socially responsive reimbursement models. In orthopedic trauma care, this is especially relevant; orthopedics-focused bundled payment models present actionable strategies for improving both care coordination and cost containment among high-risk populations. For instance, a study on Medicare’s Comprehensive Care for Joint Replacement (CJR) model demonstrated that bundled payments for hip fractures led to significant reductions in readmissions and spending without compromising care quality [61]. Similarly, a bundled care initiative targeting hip fracture arthroplasty patients led to a 13.1% reduction in 90-day episode-of-care costs and improved discharge outcomes through better coordination and reduced post-acute care use [62]. These models underscore the potential of integrated care pathways and value-based payment reforms to address the financial impact of SDoH in orthopedic trauma.

## Conclusions

This study underscores the disproportionate financial burden of orthopedic trauma among patients with SDoH. Elevated hospitalization costs, prolonged lengths of stay, and increased reliance on Medicaid highlight systemic barriers in access, care coordination, and post-acute resource use. These findings reinforce the need for more accurate SDoH documentation and greater integration of social risk data into clinical workflows. Expanding value-based payment models and bundled care initiatives that account for social complexity may be critical to advancing equitable, cost-effective care for vulnerable trauma populations.

## Appendices

**Table 4:** ICD-10-CM codes categorized under socioeconomic and psychosocial factors (CCSR Category FAC019): descriptions and classifications ICD-10-CM Code refers to the International Classification of Diseases, 10th Revision, Clinical Modification, which is used to document diagnoses and health-related conditions. The CCSR Category, or Clinical Classifications Software Refined category, developed by AHRQ, groups ICD-10-CM codes into clinically meaningful categories for research and analysis. All codes listed in this table fall under CCSR Category FAC019, which denotes socioeconomic and psychosocial factors such as housing instability, food insecurity, employment challenges, exposure to violence, and lack of social support. Source: Ref [23]

ICD-10-CM Code	ICD-10-CM Code Description	CCSR Category	CCSR Category Description
Z550	Illiteracy and low-level literacy	FAC019	Socioeconomic/psychosocial factors
Z551	Schooling unavailable and unattainable	FAC019	Socioeconomic/psychosocial factors
Z552	Failed school examinations	FAC019	Socioeconomic/psychosocial factors
Z553	Underachievement in school	FAC019	Socioeconomic/psychosocial factors
Z554	Educational maladjustment and discord with teachers and classmates	FAC019	Socioeconomic/psychosocial factors
Z555	Less than a high school diploma	FAC019	Socioeconomic/psychosocial factors
Z556	Problems related to health literacy	FAC019	Socioeconomic/psychosocial factors
Z558	Other problems related to education and literacy	FAC019	Socioeconomic/psychosocial factors
Z559	Problems related to education and literacy, unspecified	FAC019	Socioeconomic/psychosocial factors
Z560	Unemployment, unspecified	FAC019	Socioeconomic/psychosocial factors
Z561	Change of job	FAC019	Socioeconomic/psychosocial factors
Z562	Threat of job loss	FAC019	Socioeconomic/psychosocial factors
Z563	Stressful work schedule	FAC019	Socioeconomic/psychosocial factors
Z564	Discord with boss and workmates	FAC019	Socioeconomic/psychosocial factors
Z565	Uncongenial work environment	FAC019	Socioeconomic/psychosocial factors
Z566	Other physical and mental strain related to work	FAC019	Socioeconomic/psychosocial factors
Z5681	Sexual harassment on the job	FAC019	Socioeconomic/psychosocial factors
Z5682	Military deployment status	FAC019	Socioeconomic/psychosocial factors
Z5689	Other problems related to employment	FAC019	Socioeconomic/psychosocial factors
Z569	Unspecified problems related to employment	FAC019	Socioeconomic/psychosocial factors
Z570	Occupational exposure to noise	FAC019	Socioeconomic/psychosocial factors
Z571	Occupational exposure to radiation	FAC019	Socioeconomic/psychosocial factors
Z572	Occupational exposure to dust	FAC019	Socioeconomic/psychosocial factors
Z5731	Occupational exposure to environmental tobacco smoke	FAC019	Socioeconomic/psychosocial factors
Z5739	Occupational exposure to other air contaminants	FAC019	Socioeconomic/psychosocial factors
Z574	Occupational exposure to toxic agents in agriculture	FAC019	Socioeconomic/psychosocial factors
Z575	Occupational exposure to toxic agents in other industries	FAC019	Socioeconomic/psychosocial factors
Z576	Occupational exposure to extreme temperature	FAC019	Socioeconomic/psychosocial factors
Z577	Occupational exposure to vibration	FAC019	Socioeconomic/psychosocial factors
Z578	Occupational exposure to other risk factors	FAC019	Socioeconomic/psychosocial factors
Z579	Occupational exposure to unspecified risk factor	FAC019	Socioeconomic/psychosocial factors
Z586	Inadequate drinking-water supply	FAC019	Socioeconomic/psychosocial factors
Z5881	Basic services unavailable in physical environment	FAC019	Socioeconomic/psychosocial factors
Z5889	Other problems related to physical environment	FAC019	Socioeconomic/psychosocial factors
Z590	Homelessness	FAC019	Socioeconomic/psychosocial factors
Z5900	Homelessness unspecified	FAC019	Socioeconomic/psychosocial factors
Z5901	Sheltered homelessness	FAC019	Socioeconomic/psychosocial factors
Z5902	Unsheltered homelessness	FAC019	Socioeconomic/psychosocial factors
Z591	Inadequate housing	FAC019	Socioeconomic/psychosocial factors
Z5910	Inadequate housing, unspecified	FAC019	Socioeconomic/psychosocial factors
Z5911	Inadequate housing environmental temperature	FAC019	Socioeconomic/psychosocial factors
Z5912	Inadequate housing utilities	FAC019	Socioeconomic/psychosocial factors
Z5919	Other inadequate housing	FAC019	Socioeconomic/psychosocial factors
Z592	Discord with neighbors, lodgers and landlord	FAC019	Socioeconomic/psychosocial factors
Z593	Problems related to living in residential institution	FAC019	Socioeconomic/psychosocial factors
Z594	Lack of adequate food and safe drinking water	FAC019	Socioeconomic/psychosocial factors
Z5941	Food insecurity	FAC019	Socioeconomic/psychosocial factors
Z5948	Other specified lack of adequate food	FAC019	Socioeconomic/psychosocial factors
Z595	Extreme poverty	FAC019	Socioeconomic/psychosocial factors
Z596	Low income	FAC019	Socioeconomic/psychosocial factors
Z597	Insufficient social insurance and welfare support	FAC019	Socioeconomic/psychosocial factors
Z5971	Insufficient health insurance coverage	FAC019	Socioeconomic/psychosocial factors
Z5972	Insufficient welfare support	FAC019	Socioeconomic/psychosocial factors
Z598	Other problems related to housing and economic circumstances	FAC019	Socioeconomic/psychosocial factors
Z59811	Housing instability, housed, with risk of homelessness	FAC019	Socioeconomic/psychosocial factors
Z59812	Housing instability, housed, homelessness in past 12 months	FAC019	Socioeconomic/psychosocial factors
Z59819	Housing instability, housed unspecified	FAC019	Socioeconomic/psychosocial factors
Z5982	Transportation insecurity	FAC019	Socioeconomic/psychosocial factors
Z5986	Financial insecurity	FAC019	Socioeconomic/psychosocial factors
Z5987	Material hardship	FAC019	Socioeconomic/psychosocial factors
Z5989	Other problems related to housing and economic circumstances	FAC019	Socioeconomic/psychosocial factors
Z599	Problem related to housing and economic circumstances, unspecified	FAC019	Socioeconomic/psychosocial factors
Z600	Problems of adjustment to life-cycle transitions	FAC019	Socioeconomic/psychosocial factors
Z602	Problems related to living alone	FAC019	Socioeconomic/psychosocial factors
Z603	Acculturation difficulty	FAC019	Socioeconomic/psychosocial factors
Z604	Social exclusion and rejection	FAC019	Socioeconomic/psychosocial factors
Z605	Target of (perceived) adverse discrimination and persecution	FAC019	Socioeconomic/psychosocial factors
Z608	Other problems related to social environment	FAC019	Socioeconomic/psychosocial factors
Z609	Problem related to social environment, unspecified	FAC019	Socioeconomic/psychosocial factors
Z620	Inadequate parental supervision and control	FAC019	Socioeconomic/psychosocial factors
Z621	Parental overprotection	FAC019	Socioeconomic/psychosocial factors
Z6221	Child in welfare custody	FAC019	Socioeconomic/psychosocial factors
Z6222	Institutional upbringing	FAC019	Socioeconomic/psychosocial factors
Z6223	Child in custody of non-parental relative	FAC019	Socioeconomic/psychosocial factors
Z6224	Child in custody of non-relative guardian	FAC019	Socioeconomic/psychosocial factors
Z6229	Other upbringing away from parents	FAC019	Socioeconomic/psychosocial factors
Z623	Hostility towards and scapegoating of child	FAC019	Socioeconomic/psychosocial factors
Z626	Inappropriate (excessive) parental pressure	FAC019	Socioeconomic/psychosocial factors
Z62810	Personal history of physical and sexual abuse in childhood	FAC019	Socioeconomic/psychosocial factors
Z62811	Personal history of psychological abuse in childhood	FAC019	Socioeconomic/psychosocial factors
Z62812	Personal history of neglect in childhood	FAC019	Socioeconomic/psychosocial factors
Z62813	Personal history of forced labor or sexual exploitation in childhood	FAC019	Socioeconomic/psychosocial factors
Z62814	Personal history of child financial abuse	FAC019	Socioeconomic/psychosocial factors
Z62815	Personal history of intimate partner abuse in childhood	FAC019	Socioeconomic/psychosocial factors
Z62819	Personal history of unspecified abuse in childhood	FAC019	Socioeconomic/psychosocial factors
Z62820	Parent-biological child conflict	FAC019	Socioeconomic/psychosocial factors
Z62821	Parent-adopted child conflict	FAC019	Socioeconomic/psychosocial factors
Z62822	Parent-foster child conflict	FAC019	Socioeconomic/psychosocial factors
Z62823	Parent-step child conflict	FAC019	Socioeconomic/psychosocial factors
Z62831	Non-parental relative-child conflict	FAC019	Socioeconomic/psychosocial factors
Z62832	Non-relative guardian-child conflict	FAC019	Socioeconomic/psychosocial factors
Z62833	Group home staff-child conflict	FAC019	Socioeconomic/psychosocial factors
Z62890	Parent-child estrangement NEC	FAC019	Socioeconomic/psychosocial factors
Z62891	Sibling rivalry	FAC019	Socioeconomic/psychosocial factors
Z62892	Runaway [from current living environment]	FAC019	Socioeconomic/psychosocial factors
Z62898	Other specified problems related to upbringing	FAC019	Socioeconomic/psychosocial factors
Z629	Problem related to upbringing, unspecified	FAC019	Socioeconomic/psychosocial factors
Z630	Problems in relationship with spouse or partner	FAC019	Socioeconomic/psychosocial factors
Z631	Problems in relationship with in-laws	FAC019	Socioeconomic/psychosocial factors
Z6331	Absence of family member due to military deployment	FAC019	Socioeconomic/psychosocial factors
Z6332	Other absence of family member	FAC019	Socioeconomic/psychosocial factors
Z634	Disappearance and death of family member	FAC019	Socioeconomic/psychosocial factors
Z635	Disruption of family by separation and divorce	FAC019	Socioeconomic/psychosocial factors
Z636	Dependent relative needing care at home	FAC019	Socioeconomic/psychosocial factors
Z6371	Stress on family due to return of family member from military deployment	FAC019	Socioeconomic/psychosocial factors
Z6372	Alcoholism and drug addiction in family	FAC019	Socioeconomic/psychosocial factors
Z6379	Other stressful life events affecting family and household	FAC019	Socioeconomic/psychosocial factors
Z638	Other specified problems related to primary support group	FAC019	Socioeconomic/psychosocial factors
Z639	Problem related to primary support group, unspecified	FAC019	Socioeconomic/psychosocial factors
Z640	Problems related to unwanted pregnancy	FAC019	Socioeconomic/psychosocial factors
Z641	Problems related to multiparity	FAC019	Socioeconomic/psychosocial factors
Z644	Discord with counselors	FAC019	Socioeconomic/psychosocial factors
Z650	Conviction in civil and criminal proceedings without imprisonment	FAC019	Socioeconomic/psychosocial factors
Z651	Imprisonment and other incarceration	FAC019	Socioeconomic/psychosocial factors
Z652	Problems related to release from prison	FAC019	Socioeconomic/psychosocial factors
Z653	Problems related to other legal circumstances	FAC019	Socioeconomic/psychosocial factors
Z654	Victim of crime and terrorism	FAC019	Socioeconomic/psychosocial factors
Z655	Exposure to disaster, war and other hostilities	FAC019	Socioeconomic/psychosocial factors
Z658	Other specified problems related to psychosocial circumstances	FAC019	Socioeconomic/psychosocial factors
Z659	Problem related to unspecified psychosocial circumstances	FAC019	Socioeconomic/psychosocial factors
